# “It Can Be Magic:” Creating Age Awareness Through Contemporary Dance

**DOI:** 10.3389/fspor.2022.795541

**Published:** 2022-03-10

**Authors:** Pirkko Markula, Kathy Metzger, Tamara Bliss, Wendy Gervais, Mary Ann Rintoul, Jodie Vandkerkhove

**Affiliations:** Faculty of Kinesiology, Sport, and Recreation, University of Alberta, Edmonton, AB, Canada

**Keywords:** aging, contemporary dance, performance, memory-work, women

## Abstract

Professional dancers typically retire before age 40. Although the physical requirements for dance performance are often considered the reason for retirement, there is an increasing number dance researchers who demonstrate that the idealization of youthfulness on the stage is also a result of complex cultural, social, and economic realities and as such, in need of critique. As a group of mature women dancers who continue to perform, we aim to critique the idealization of youthfulness as a form of ageism in professional dance. In this paper, we present findings from our feminist memory-work study in which we critically reflected the rehearsals and performance of a choreography titled “Initiation.” We detail three main themes—”It Will Only Get Worse;” “It Can Be Magic;” “Once a Dancer Always a Dancer”—that emerged from our study. We conclude that we gained critical awareness of the gendered and ageist construction of dance as a performing art. As a result, we now feel empowered to continue our work as mature dance artists.

## Introduction

Dance has recently been offered an attractive physical activity option for older women who tend to be less physically active than older men, yet enjoy the fitness gains, socialness, and movement aesthetics of dance (e.g., Cooper and Thomas, 2002). Provided these benefits attached to dance for aging women, it is not surprising that the majority of research focuses on the physiological and psychological benefits usually obtained in recreational social dance (e.g., Judge, 2003; McKinley et al., 2008; Zhang et al., 2008; Eyigor et al., 2009; Hui et al., 2009; Keogh et al., 2009; Sofianidis et al., 2009; Dewhurst et al., 2014; Marks, 2016; Merom et al., 2016). While these findings emphasize the importance of the “social” health benefits of dance, there is less socio-cultural research on ageism and its impact on mature women's experiences of their activity. In this paper, we explore, from a feminist perspective, the experiences of a group of mature women who returned to perform contemporary dance to defy ageism in this cultural context. We will first consider the relevant literature on the “idealization of youthfulness” in western theater dance that renders mature dancers invisible or even unsuitable to perform. We will then detail the memory-work method that we used to explicate our feminist approach to our dance practice. Finally, we will discuss how the process of choreographing and performing contemporary dance emerged as a potential subversion of ageism from our memory-work sessions.

## Professional Dance and Idealization of Youthfulness

The ideal image for a professional woman dancer in Western theatrical dance is often a young, poised, and slender body performing extreme feats of balance and flexibility. Faced with this dominant imagery, few professional dancers continue their careers beyond the age of 40. Several dance scholars have argued that the dancing body is socially constructed as “aged” in its late 30s, rendering mature dancers unsuitable as performers (Wainwright and Turner, 2004, 2006; Schwaiger, 2005, 2012; Coupland, 2013; Brandstetter and Nakajima, 2017; Martin, 2017). Schwaiger (2012), for example, noted that professional dance reflects the general devaluation of older age in Western cultures. As “clearly a body-based domain,” she continued, dance is open to the idealization of youthfulness (p. 1). In this context, where many dancers face intense competition and pressure to conform to gendered body ideals, “an intensification and acceleration” of aging is prevalent (p. 1). This intensification, Schwaiger concluded, “highlights the social constraints impinging on [dancers'] social recognition and cultural valorization, which are culturally naturalized and hidden” (p. 1). The expectation of ballet and contemporary dance culture as a young people's affair is so pervasive that dance studies scholars have recently initiated further discussion regarding the stereotypes of aging in professional dance.

Wainwright and Turner's (2004, 2006) research on the Royal Ballet in London, UK, where they interviewed 11 ex-dancers, supports Schwaiger's (2012) observations. Professional ballet dancers who had built their lives around being physically fit often found their aging bodies to be “decrepit,” “deteriorating,” and even “scary” and retirement from dance “demoralizing” and “traumatic” (Wainwright and Turner, 2004, pp. 100–118). Using Bourdieu's concept of habitus, the researchers found the retirement process from a career in a culture of “youthful aging” (a 30-year old is considered old), “‘a cruel business' indeed” (Wainwright and Turner, 2006, p. 247). The dancers also believed that the audience does not want to see sub-standard performances (by aging bodies) and thus, considered older dancers insisting on performing “sad” or “mentally sick” (Wainwright and Turner, 2004).

Dance studies researchers adopting a critical age perspective further demonstrate that the requirement for “empirical youthfulness” on the stage is a result of complex cultural, social, and economic realities in need of critique (Martin, 2017, p. 17). Consequently, they argue for further examinations of how dance can also “reconfigure and challenge dominant understandings of age(ing)” (Martin, 2017, p. 87). Contemporary dance, particularly, can act as a tool for creating critical age consciousness in society at large by exposing, questioning, and subverting stereotypical representations of aging bodies (e.g., Albright, 2010; Nakajima, 2011; Schwaiger, 2012; Martin, 2017). Martin (2017) concludes that it “can be a site to question and widen our ideas on age, age appropriate behavior, and appropriate images of bodies of different ages” (p. 74). Anna Halprin, Yvonne Rainer, and Twyla Tharp exemplify contemporary dancers who, continuing to perform well beyond age 70, have established different aesthetic potentials for their continually changing and evolving bodies (e.g., Schwaiger, 2012; Brandstetter and Nakajima, 2017; Martin, 2017). To subvert aging stereotypes, contemporary dance, however, needs to acquire a different aesthetic sensibility and a different appreciation of ability “not as a loss, but as a gain” (Brandstetter and Nakajima, 2017, p. 3). Some researchers have explored, empirically, the possibilities for such “age appropriate” dance strategies.

In her study, Coupland (2013) captured older (42–72 years old) contemporary dancers' experiences in dance classes and a final performance of their company, *Dance Innovations*, in the UK. This company openly located itself in opposition to the “restrictive discourse of youth, dance and watchability” (p. 8). Through class participation, field notes, focus groups, and dancers' diaries, Coupland discovered that “even in a self-consciously age-emancipating institution as Dance Innovations, informants are frequently aware of the potentially repressive ideology of mirrored old age” (p. 20).

Schwaiger (2005, 2012) explored similar themes cross-culturally through semi-structured life history interviews with 30 professional dancers from various theatrical dance backgrounds between the ages of 27–76 in Australia, India, Germany, Ireland, and the Netherlands. Like Coupland's (2013) dancers, they were strongly influenced by the dominant cultural ageist discourse, but also subverted it by continuing to perform. These dancers chose less “codified” dance forms to establish a mature presence on the stage instead of relying on such “athletic” movement vocabulary as multiple pirouettes or jumps. To perform “their own age,” the dancers further engaged in regimes of self-management with a healthy diet. Unlike Wainwright and Turner's (2004, 2006) ballet dancers, these dancers did not necessarily find physical aging resulting in retirement that was more to do with increased family commitments, reduced performance opportunities, or lack of dancing peers in midlife. While some continued to perform as independent artists, they found their art undervalued compared to more mainstream works presented by professional companies.

In their study, Southcott and Joseph (2020) interviewed six Australian contemporary dancers and their teacher who had formed a dance group *Fine Lines* to continue dance practice and performance beyond age 40. Combining Bourdieu's concept of habitus and an interpretive phenomenological analysis (IPA), they found that the contemporary dancing body became a site of self-creation, self-realization, and wellbeing. The dancers celebrated their maturity by developing new skills, learning through improvisation, and maintaining a sense of their dancing selves in a weekly technique class as well as regular performances. With a lifetime of dance training, they continued to challenge themselves albeit taking care of their bodies to maintain their health and wellbeing. Having a similar dance background created a feeling of community where the dancers could replenish and extend their commitment to dance. The researchers conclude that “older ballet and contemporary dancers should be recognized and valued as vibrant artists who contribute to their local and wider community” (p. 606).

This small body of dance studies research illustrates how social constructions of maturity, physicality, and gender can constrain dancers' continued enjoyment of dancing and wellbeing in later life. The findings further demonstrate how contemporary women dancers, in particular, can resist the social construction of the aging body as frail, weak, and unsuitable for public performance. Our research, inspired by the critical dance studies scholars, continues to highlight dance as a site for the subversion of the cultural norms of youthfulness through the experiences of mature women dancers who continue to perform. It expands the previous literature on aging and contemporary dance by employing a feminist methodology that reduces the researcher-participant hierarchy by placing all the participants as co-researchers. Such a methodology is absent in the previous accounts of mature women's dance experiences.

## Methodology

Our research project centers around a contemporary dance group *Initial 6*, made up of six accomplished Canadian contemporary women dancers, mature in their years of training, teaching, and performing, who originally came together to be able to continue performing with like-minded professionals. The *Initial 6* members are trained in dance at the University level with three members with degrees in dance. Now aged between 42 and 65 years, we had previously performed in various “pick-up” contemporary dance companies that came together when funding for staging a performance was available. Only one of us had performed as a professional with the same company for several years. Although retired as performers, three dancers still obtained fulltime living as dance teachers and choreographers at the time of the research. The other dancers had careers in education. This research was based on our performance in the University of Alberta's dance group Orchesis's annual Motif performance in 2015 in Edmonton, Canada. Having the performance as our main goal at this point, we began rehearsing the choreography 9 months before entering the stage. While our rehearsal schedules were flexible to accommodate each dancer's time table, we generally rehearsed 2–3 h each week with slightly more frequent rehearsals nearer the performance date. After the performance, we wanted to reflect our experiences of re-entering the stage and chose memory-work as our research method. Before embarking on our project, we obtained ethical approval from the University Alberta Ethics Board.

### Memory-Work as Feminist Method

We used memory-work as a method to reflect upon our performance experience and to theorize about dance as a bodily expression of our mature “selves-as-subjects” (Schwaiger, 2005, p. 116). Memory-work was developed in the 1980s by a group of German women who, drawing on Marxist feminism, wanted to research female sexualization (Haug, 1987; Stephenson, 2005). In addition, they aimed to destabilize the power relations between an outsider (objective) researcher and the subjective researched by placing themselves both as researchers and researched of their own work (Stephenson, 2005). The subjective dimension included an investigation of their own experiences that the women then theorized collectively in a group. Memory-work, thus, attempts “to question the connections between experience and selves or subjects, to simultaneously envisage experience as socially produced and amenable to reinterpretation” (Stephenson, 2005, p. 34). The experience, nevertheless, is understood, not as evidence that “exist[s] ‘out there', ready to be picked up and inserted into an analytical or interpretative process” (Jansson et al., 2008, p. 229), but to serve as a starting point for the investigation (Stephenson, 2005).

At the same time, experiences do not come into existence outside social and political contexts (Jansson et al., 2008). Consequently, Haug (1987) emphasized that new understandings of social structures can be reached only by theorizing experiences. Memory-work, therefore, is intended to have explicit political value: it is to enable interventions that, in addition to documenting the problematic processes, is to initiate social change. It is a method that “may provide one way to get hold of the political in the lived practices and experiences of everyday life” (Jansson et al., 2008, p. 230). In memory-work, individual women, instead of passively being determined by their social contexts, can actively appropriate social structures and in doing so can become who they are. As Stephenson (2005, p. 35) summarized: “Haug and others were interested in ‘experience as [the] lived practice… of a self-constructed identity… [as something] structured by expectations, norms and values… [which] still contains an element of resistance, a germ of oppositional cultural activity”' (1987, p. 42).

In summary, memory-work explores and theorizes “how individuals construct themselves into existing social and power relations” (Jansson et al., 2008, p. 231) and as such how individuals establish and reproduce the social order in the lived practices of everyday life. Memory-work is a collective method: all participants contribute equally to the creation of the empirical material as well as its analysis and interpretation. This effectively blurs the distinction between the researcher and the research subjects and as such, “stands in stark contrast to the hierarchical and individualistic academic culture” (Jansson et al., 2008, p. 231). In our research project, accordingly, all participating *Initial 6* dancers were considered as equally contributing co-researchers. Following Jansson et al. (2008) we used memory-work as a tool “to unmask and challenge deeply naturalized” social constructions of aging and femininity in dance (p. 238). By explicating what “appeared as self-evident, natural, and/or personal” (Jansson et al., 2008, p. 238), we aimed to unravel the possibilities for agency to question and challenge social relations in dance that uphold compulsory youthfulness. As we examined the possibilities to disrupt social categories of gender and age in contemporary dance through our lived performance experiences, the understanding of the aging, feminine dancing self, then, arose, intersubjectively, through interaction with a group of other dancers.

As a feminist collaborative method, memory-work consists of writing individual memories and discussing them in a group setting. Before gathering to the group, the co-researchers, in collaboration, compile “triggers” for each memory (Onyx and Small, 2001). To do this, we met as a group to more closely familiarize ourselves with memory-work as a research method. In this meeting, we brainstormed the types of “topics” about which we wanted to write. Based on this discussion, we acquired five triggers from rehearsing and performing together in “Initiation.” They were: “When I just could not remember the movement;” “I cannot do this because…”; “Rehearsing with the group of experienced dancers;” “Feeling it;” and “The Performance.”

Based on each trigger, the co-researchers wrote detailed pre-prepared memories without interpretation or autobiography for the group discussion. Memory-work provides clear guidelines for writing each memory (Crawford et al., 1992) that we also followed. First, each memory should include a specific action or event inspired by the previously defined triggers. Second, each memory should be written in third person. This writing technique is to invite each co-researcher to observe aspects of themselves (e.g., aging) without having to justify or affirm the practice. In addition, it facilitates standing back from the actions the co-researchers describe (Stephenson, 2005) to assume the positions of “‘she' of the written memory and the ‘I' of the group discussion” (Stephenson, 2005, p. 37). Finally, the memories should include as much detail as possible but without interpretation or explanation (Crawford et al., 1992; Stephenson, 2005). Including details, even if seemingly inconsequential or trivial, is important but any explanations should be avoided as these are solicited in the later group meetings as shared interpretations and theorizations of the memories (Stephenson, 2005). We distributed our memories to the other co-researchers *via* email before each group meeting. We had five group meetings to discuss the memories initiated by the five pre-determined triggers.

Several researchers have suggested guidelines for analyzing the memories in the group sessions (Crawford et al., 1992; Onyx and Small, 2001; Markula and Friend, 2005; Stephenson, 2005; Jansson et al., 2008; Markula and Silk, 2011). Generally, these include the following steps:

1. Each memory is read by its author after which each co-researcher expresses ideas about each memory in turn;
2. The group looks for similarities and differences between the memories and looks for further clarifications to the events which are not readily understandable;
3. The group identifies clichés, generalizations, contradictions, or cultural imperatives in the share memories;
4. The group discusses popular conceptions, sayings and images about the topic;
5. The group examines what is not written in the memories but what might be expected to be included in them (adapted from Stephenson, 2005).

Our group discussions were based on a similar order. In the first round of discussion, each co-researcher read their memory after which the others expressed their ideas about it. In the second round, the group looked for similarities and differences between the memories and also asked questions of details or events that were not clear based on the memory. Based on these rounds, the group then identified generalities, contradictions, and cultural influences that connected the memories to popular conceptions and images of aging, femininity, and dance. The group further searched for possible silences or absences in the memories. These were aspects that the co-researchers assumed should have been included in the memory. For example, we discussed how the cultural ideals of femininity may have impacted events around self-doubt even if details of this dimension were not included in the memory. The five group discussions were audiotaped and then transcribed by one of the co-researchers.

Although all *Initial 6* members contributed equally to the memory-work process, the findings were analyzed by the two first authors with the permission of the others to use the work for further publications. Although the interpretation of the memories is embedded in the memory-work process when the co-researchers make linkages to other memories (Stephenson, 2005; Jansson et al., 2008), these authors provided the final theorization phase of memory-work in which the insights from the memories were related to wider academic literature. They used the written memories as well as the transcripts from the group discussion to analyze the data through an interpretive and critical theory framework of the memory-work. This framework also provided tools to carefully acknowledge the complexities of being both co-researchers and participants in the analysis of the memory-work material: as memory-work researchers we were interested in self-constructed identity at which the co-researchers actively arrive through shared experiences that are both personal and political; individual and public (Stephenson, 2005).

The *Initial 6* co-researchers have chosen to appear by their actual names in the findings section. We now present themes that capture the main similarities, but also embed differences and contradictions in our identity constructions. We also point to the cultural images of aging to further theorize how our identities as dancers were actively constructed through our rehearsal process and performance.

## Findings

The major themes that emerged from the memory-work sessions were “It Will Only Get Worse;” “It Can Be Magic;” and “Once a Dancer Always a Dancer.” Each theme had several subthemes that reflected our growing critical awareness of the social construction of aging and gender in dance unraveling through our discussions. We now consider each theme in more detail.

### It Will Only Get Worse

This memory-work theme has two interrelated sub-themes which reflect our attitudes prior to and at the beginning of the choreographic process. These themes stemmed from the current cultural and social context of aging and as such, tended to reproduce established ageist ideas of the feminine dancing body.

#### Fear and Negative Self-Talk

Several *Initial 6* dancers shared their fears and doubts about starting to rehearse for a public performance that many dancers at our age no longer consider appropriate (e.g., Wainwright and Turner, 2006). Many of us also found committing to regular rehearsals no longer feasible at the current stages of our lives. Jodie recalled when asked about her interest in joining the group:

Too busy, I am a mom, instructor, as well, a grad student, when am I going to fit this into my time table of life while trying to maintain “balance” …, I really feel like I am in survival mode, is adding another thing going to make me even more tired.

As a mom, Jodie's “survival mode” when battling with finding a “balance” also reflected time demands affecting particularly women dancers who, at this stage of their lives, continually negotiate demands between their family, their professional lives, and dance commitments. Similar to Jodie, many women dancers at this stage prioritize their children over their careers (Schwaiger, 2012). Although not having to negotiate similar time constraints, Wendy recalled actually resenting the time-consuming rehearsals at the beginning: “When I thought about going to rehearsal, I sometimes resented the rehearsal: the time it took, the rearranging of schedules.” Most of all, however, she resented her “aging and misbehaving body.” Wendy further wrote that, psychologically, she could not “do this dance because I worry” and she “hated” to worry. Wendy's reflections of her aging dancer's subjectivity aligned with the traditional view in which the body becomes defined “through loss of the attributes of youth” (Schwaiger, 2005, p. 116): the body that the dancer used be able to control without extensive “worry.” To accommodate such concerns, we started rehearsing early−9 months before the performance which is an unusually generous timeline for many contemporary dance groups—to get used to dancing again and to accommodate our timetables.

Feeling time pressure, similar to the dancers in Schwaiger's (2012) research, was a factor for some dancers. There were added fears about the physical demands of dancing again. For example, Mary Ann, who was very aware of the fact that she “had not been dancing regularly, had not taken classes for years and certainly had not performed for at least 15 years” was afraid of not being able to “physically keep up with the other dancers” and anticipated being “miles behind the others.” These factors made her question “whether or not I could…get on stage to dance.” In fact, many of us were worried about the prospect of a public performance. For example, Jodie, the youngest of the co-researchers, anticipated not being as skilled as the other, more mature dancers and consequently, worried about appearing on stage:

I do not want to look out of place. I am an amazon on stage. Too out of the loop, I have not been on a traditional stage for so long; I don't have the skills to perform like this anymore. I may hit a light or not know my surroundings.

Jodie is referring to her tall body that, as she believed, made her stand out, too conspicuously, from the rest of the group. Unlike the “physically very fit” dancers in *Fine Lines* (Southcott and Joseph, 2020, p. 600) who had taken class and danced together for several years, we had also other body size related negative reflections. Mary Ann believed that she had gained weight as did Kathy who felt too embarrassed to even talk about her body size:

Because I was overweight…but not my ideal body that I like to be, as fit as I usually am. So at the time, when I look back at some of those videos, I feel I am still dancing well, but I don't look that way… so I am a little hard on myself, so I didn't talk about it because I was in denial or embarrassed.

Tamara, a former professional dancer, recalled her desire to return to her “performance weight” after some weight gain due to drugs that she was prescribed for her back pain.

As far as body image, I had issues because, I had been on…drugs before…and one of the side effects of the horrible drug for my back was weight gain, constipation…and I really wanted to lose the weight…So we talked about doing this piece, well I have to get in shape, and have to lose this weight. My goal. So I made sure that I went on Weight Watchers…and I wanted to be, my performance weight was 118lbs, and I made it.

In addition to the drug, Tamara, as well as Kathy, attributed weight gain to menopause during which “hormones are all over the place” (Kathy). In the following exchange, they consider such weight gain detrimental both in terms of how it looks on the dancer's body and how it impedes performance:

Tamara: It is harder to move, or get your leg up by your ear when you weigh a couple pounds more.Kathy: Yah the tissue is in the way.Tamara: Yah come on, what is that roll?

Pirkko recalled that the requirement of the thin dancing body, instilled on her during her dance training, was very difficult to let go, particularly when the body is aging and one still wants to appear on stage.

At this point, we appeared to take the requirements of an ideal feminine dancing body for granted without assessing them, from critical feminist perspective, as socially constructed, cultural constraints prevalent in dance (e.g., Schwaiger, 2005). As Jodie indicated, we were also quite anxious about our aging bodies that, we worried, would not be able to perform at our previous level. At this point, it was not so much about “the inevitable decline” of the aging body (Southcott and Joseph, 2020, p. 601), but the uncomfortable feeling of looking like untrained dancers and the “unwatchability” (Coupland, 2013) of such feminine bodies on stage. Our memories also reflected the deeply rooted fear of deviating from the gendered feminine ideal dancing body to publicly display our flaws now exacerbated with the “devastating evidence of aging” (Foster, 1997, p. 598). Unlike the *Fine Line* dancers (Southcott and Joseph, 2020) who felt that performing as an older person was “less pressured” and “a huge relief” (p. 8), we hesitated to perform after retirement. The *Fine Lines* company also trained in a regular weekly technique class (Southcott and Joseph, 2020) that we had not a chance to organize. Without an opportunity to train “privately” in a studio, we were possibly even more anxious about performing in public. Pirkko summarized that she was “only feeling tired, anxious, and nervous. And disappointed with my feelings. I am too old for this” even if she tried to convince herself that it no longer “matters” if one's performance is not perfect. Her negative conclusion mirrors the cultural valorization of youthfulness in dance in which expressiveness by a mature body is no longer valued (Schwaiger, 2012).

#### Awareness of Injuries

Common to dancers of our age (e.g., Wainwright and Turner, 2006; Southcott and Joseph, 2020), many of us now nurtured previous injuries that had changed our understandings of our abilities as dancers. For example, Mary Ann had an old injury to her left toe joint and Tamara had a back injury that now significantly shaped her understanding of herself as a dancer. She explained:

What I can do at the age of 60, is now quite a bit different than what I could do at 30 or 40. At nearly 63 years of age, I now hear myself saying…reasons mostly related to the pains and aches my body is feeling:

“I cannot do this because my back does not let me move that way.”
“I cannot do this because I have intense scar tissue in that hamstring.”
“I cannot do this because I experience vertigo when I tilt my head in that direction.”
“I cannot do this because my hands have no strength due to my arthritic thumbs.” “I cannot do this because my body just will not let me anymore!”

Consequently, Tamara recognized that her bodily ability, instead of mind, now dictated her performance aptitude: “The ultimate defiance for a dancer is a body that betrays one's will, where one's will always led the body.” Tamara's comment also reflects a hierarchical relationship where her body was to learn to perform under the control of her mind (Schwaiger, 2012). This dualism was breaking due to her aging body that had now betrayed Tamara's dancing self and contributed to her feelings of inevitable growing old. If our earlier negative self-talk referred to our unfit looking bodies, Tamara's perception her physical decline echoed the attitudes by the *Fine Lines* dancers in Southcott and Joseph's (2020) study who also had to carefully “manage their bodies” (p. 601) when it is was no longer possible to make them do what the dancer wanted. Schwaiger (2012) noted that such body management, nevertheless, enabled the aging dancers in her study to continue their performance careers. At this stage of our memory-work reflection process, we also revealed that similar body management could get us through the performance.

Pirkko gained an injury during the rehearsal process. She recalled one rehearsal during which there was a stabbing pain in my right foot, so bad that I had to stop. I do not usually stop in the middle of the piece. I felt like crying—not so much because of the pain, but because of the feeling that I now cannot do the piece! What was this pain anyway? On the bottom of my foot? Will it go away? Will I ever jump again?

Similar to younger contemporary dancers (Markula, 2015), Pirkko was most concerned of not being able to perform. Instead of attending to the possible causes of the injury or resting, Pirkko just “added more and more tape” and ended up performing with shoes when the others danced bare foot. Despite being a mature dancer, her defiance to injury and her “performing at all cost” approach reflected typical attitudes among younger professional and pre-professional dancers (e.g., Wainwright and Turner, 2006; McEwen and Young, 2011; Markula, 2015). Wendy, who despite a broken toe had continued to dance earlier in her career, had a different view of injuries now. She acknowledged that “[w]e dancers often pride ourselves in continuing to dance through injuries, prolonging the injury and risking so much,” but now questioned “why we do this. I like to think that I do take better care as I age. I want to dance for my whole life.” While rather unforgiving to her own injured body, even Pirkko who choreographed the piece, was careful to adjust the rehearsals to accommodate the other dancers' physical conditions. She reflected on the overall picture of the dancers' physical issues on a rehearsal day:

Tamara['s] back is troubling her after yesterday's Garuda class. Wendy approaches me to warn that her hamstring is acting up—she could not sleep much last night…It turns out that Jodie was also pushed by a car and fell when biking and her knee is all black and blue. I am trying to endorse that they all need to be careful and mindful of their injuries.

Her attitude here aligned with the approach by the *Fine Lines* dancers who carefully looked after their bodies during their training (Southcott and Joseph, 2020).

At this point of the interpretive memory work process (Jansson et al., 2008), we, similar to the *Dance Innovation* dancers (Coupland, 2013), reflected our identities as dancers against our earlier notions of what it meant to be a performing dance artist. Our concerns of weight gain, lack of physical stamina and skill mirrored the idea of empirical youthfulness—the social idea that dance is only for young bodies—that affected our assessments of what is expected to dancers who publicly perform dance (Schwaiger, 2005; Coupland, 2013; Martin, 2017). In this context, older dancers, particularly women, can be deemed as “unwatchable” (Wainwright and Turner, 2004; Coupland, 2013). Our memories further illustrated an underlying belief that as mature dancers, possibly without the same physical capacity as we had in our earlier careers, we were expected to withdraw from the public gaze. Consequently, our negative self-talk reflected some of the ageist attitudes (e.g., Wainwright and Turner, 2004; Schwaiger, 2012; Brandstetter and Nakajima, 2017; Martin, 2017) in contemporary dance: we expected our bodies to conform with the gendered, thin idea of the high-performance dance body and we were afraid of no longer having the appropriate “social recognition and cultural valorization” of contemporary dancers at our age (Schwaiger, 2012, p. 1). Different conceptions of ourselves as dancers, however, began to evolve during the rehearsal process and the performance. These are evident in the next theme that emerged from our memory-work discussions.

### It Can Be Magic

This second theme emerged as we, during further discussion and analysis, began to challenge what appeared as personal experience (Jansson et al., 2008) to unmask the social construction of aging in dance. We now entered a process of unraveling possibilities to question social relations that uphold the empirical youthfulness in dance to alternative, subversive readings of aging and dance (Schwaiger, 2005). Our first subversive reading drew on the pleasure of working with mature dancers who, while not confirming with the stereotypical youthful feminine bodies on stage, provided a supportive and constructive environment for dance making.

#### The Joy to Dance With Women of Experience

Parallel to the *Fine Line* dancers (Southcott and Joseph, 2020), we echoed the positives of working together as a group of mature, women dancers who respected each other, took responsibility for and were deeply committed to the project, and created a supportive and collaborative environment. As Jodie described our group:

Blessed, committed, hungry, driven, self-motivated, and happy; all of these words can express the process of rehearsing with the group of experienced dancers…This was a blessing; to be surrounded by these experienced dancers.

Based on Kathy's experience, these characteristics can be uncommon when working with younger, inexperienced dancers. Wendy also remembered that we were a possible exception to many other rehearsals she had attended because

[w]e spent most rehearsal time actually dancing. That was the best! Socializing was never discouraged. Because we dancers are mature and motivated (for like and various reasons), we intuitively ordered our rehearsals to be successful.

Schwaiger (2012) suggested that this type of “intersubjectivity” increases with maturity when “older dancers tend to become more associated with others than dancers who are younger” (p. 62). Our experiences with dancing with other women with similar age and ability aligned with Schwaiger's observation.

The *Initial 6* dancers took responsibility for their own learning by “marking the previous choreography gently exchanging differing opinions about the correct timing” (Pirkko) to be prepared for the actual rehearsals. Confident and supportive, the emotionally mature dancers respected and drew inspiration from each other. Tamara recalled:

When partnering movement with Jodie, I remember sensing her timing, impulses and breathing and matching or weaving with her energies, my movement impulses. It is a treat to be able to dance this way with others. To me, it feels as if my body and its energies extend beyond the obvious physical limits.

It was evident that Tamara who previously talked about the many physical limitations of her body, now, inspired by the other dancers, found new physicality. Instead of her earlier dualist mind-body relation, Tamara now described what Schwaiger (2012) defined as embodied relationship in which thinking, feeling, sensing, and moving are connected with other bodies. Several co-researchers wrote about commitment, “genuine interest” (Pirkko), and investment in the choreography “with a full-on discussion with each section as it has become a very democratic process, everyone giving their two-cents” (Kathy). Jodie summarized: “First rehearsal…you could see, feel and smell the level of commitment from the experienced dancers, it was refreshing, exciting, and exhilarating to be a part of this process…I felt the team.” Similar to the *Fine Lines* mature dancers in Southcott and Joseph (2020) study, we sensed a feeling of community of contemporary mature dancers with similar dance backgrounds and shared process of building the choreography. In this community, it was possible to replenish and revitalize our commitment to dance.

In addition to trust and respect, working together with other mature dancers elicited enjoyment. Several co-researchers reflected on the feeling of joy for dancing. Kathy explained:

For me the joy is really about this amazing process with the other experienced dancers, learning a dance, being in our bodies, all being mature and inspiring women!

Tamara echoed:

My performance reflection touches on feelings of joy, fun and…actually dancing the dance…absorbed in the movement.

Mary Ann added:

When I recall the entire experience of rehearsing with this particular group of dancers, I can't help but smile. In essence, this was the joy of the whole thing for me, to dance with women of experience…We all come to the rehearsal with our strengths and limitations, but with great enthusiasm and joy!

Jodie and Wendy similarly attributed their joy to working with a group of experienced, “seasoned” (Wendy) dancers. The pleasure of dancing has been reported also by several other researchers (Kolb and Kalogeropoulou, 2012; Ali-Haapala et al., 2020). In these studies, the joy derived from the physical and cognitive challenges in dance classes that could include participants with various levels of dance experience. We felt joy at being able to dance with others at a similar level of technical training that then provided an appropriate physical challenge to “be in our bodies” and feeling the dance movement again. As Tamara summarized: “[m]y performance reflection touches on feelings of joy, fun and…actually dancing the dance…absorbed in the movement.” Mary Ann aptly concluded that “[w]e shared a powerful experience of the dance, and common identity as dancers regardless of age, ability or performance history. This was a wonderful feeling.”

#### Trust Your Body, You Know the Movement

In addition to enjoyment of working with mature, competent dancers, we began to reflect upon the possibilities of our mature bodies to resist the idea that only young bodies are suitable for performing dance. According to previous research, the aging feminine body in dance can be considered physically unable to reach the standards for public performance (e.g., Wainwright and Turner, 2004; Schwaiger, 2005; Coupland, 2013; Southcott and Joseph, 2020). However, the dancers in Coupland's (2013) study “found their bodies” again in dance. Similarly, we “moved back into our bodies” as the rehearsals proceeded. Although Wendy initially resented attending the rehearsals, she gained back her love of dancing: “But once you get started you realize that you love what you are doing and you love the inclusion and the passion of those around you.” Other dancers began to appreciate their current bodies. Tamara, who had earlier indicated her body letting her down, now explained that “[h]aving danced for 59 years of my life, and certainly 49 dynamically, capably and professionally for 30 of those, I will continue to find ways that I can move. I cannot do this exactly but I can [do] this.” Wendy added that there can even be benefits of maturity, because, “as we age…we are able to successfully deal with some things that make us uncomfortable.”

The mature dancers in Schwaiger (2012) study became more confident when they invested in their performance presence and emotional expression rather than physical, technical ability. To value our physicality, we also modified the choreography to favorably display our capable bodies. Unlike the *Fine Lines* dancers (Southcott and Joseph, 2020), however, we did not consider our physicality as a restriction, but as an artistic opportunity:

It was, of course, necessary to respect our bodies, but rather than thinking of them as limitations, creating movement that was modifiable for multiple bodies, yet allowed us to move together as a group, provided many important creative moments. (Pirkko)

*Fine Lines* dancers also continually sought “micro” movements to accommodate their aging bodies (Southcott and Joseph, 2020). Similarly, Schwaiger (2012) found mature dancers resorting to “smaller” movements to “perform their age” (p. 76). This derived from dancers wanting to avoid the assumed ridicule and audience criticism when appearing on the stage that is usually reserved for youthful physicality. Our intention differed from such “age-appropriateness” (Schwaiger, 2012, p. 76). Although the choreography accommodated our aging bodies, our modified movement vocabulary was not designed for decreased physical demands *per se*. As highly technically trained dancers, we wanted a performance that challenged the notion of mature dancers capable of less physical expression than young dancers. As Wendy put it: “I loved the intension of our movement and that the choreography allowed that.” As the choreographer, Pirkko repeated many times that she did not want “pity claps” at the end of the performance. Her intention as the choreographer was also to connect to the audience, at times, with certain defiance: with a direct stare frozen in a pose that she described “as a really ugly movement”—a challenge, a “flirt” through which we expressed “an attitude” (Kathy), an “intimidation” (Tamara). We aimed for a growing awareness and connection with the audience that we, the confident mature dancers, now were able to exhibit in addition to solid technical ability. Schwaiger (2012) reported mature dancers aiming for increased “stage presence.” This, however, was more often commented on by the male dancers whereas the women dancers found such confidence more difficult to achieve in the dance world where women often receive harsher criticism. In our case, performing as a group of mature dancers who supported each other, potentially increased our confidence to command stage presence and defy the ageist notions of the dancing femininity.

Although we originally were apprehensive regarding our physical ability to sustain the demands of the rehearsals and even more hesitant to perform, only a few of us reflected upon actual physical problems during the process. Wendy described the cramps that hit her in the middle of the performance and Pirkko wrote about her recurring foot problem that prevented her from dancing in bare feet. For Mary Ann, however, “[a]ll time stood still…body aches were undetectable” in the performance. Most of us recalled the joy of moving and performing in a “flow” like state or as Mary Ann described it, being “somewhat like an athlete ‘in the zone'…, or a child ‘in the zone' as they play fervently.” Tamara visually depicted similar “sublime” experience (Schwaiger, 2012, p. 61) in her dancing as a flow of words:

“My own years of experience came back to me yet again to carry over into this fleeting time of existence on the stage.

Floating,

turning,

gliding,

jumping,

falling,

pushing,

flowing,

stretching,

zinging…

…breathing hard and sweating are just some of the ways I remember dancing.”

These embodied experiences were “timeless” and “ageless:” not limited to young bodies, we came back to being “athletes” and dancers playing “fervently” again. Being able to articulate these experiences through our memory-work discussions indicated a further subversion of agism in dance culture.

In addition to moments of exhilaration, both Kathy and Wendy experienced meditation like quietness in performance where one concentrated “on one movement, one breath at the time” (Kathy). These moments indicated further resistance to the ageist notion of youthfulness in dance in several ways. When Wendy described “dancing in the moment,” this moment provided “quiet in mind” that she cherished as calmness in her life. Wendy's reflection here differs from the *Dance Innovation* dancers' refocus on mindfulness to forget about their aging bodies and their “unwatchability.” For her, dancing offered a welcome quiet space from the buzz of her everyday life (Coupland, 2013). Instead of subscribing to the ageist notion that old people should live quietly, Wendy sought increased vitality through her focus on dancing in the moment. When Kathy now reflected on her experiences of dancing in the moment, she compared herself to her younger dancer's self in positive terms:

It felt like I use to…when I was younger and in shape. I noticed that my muscles and joints felt strong without any pain. I was feeling like I could control my movements with ease as I was warming up.

In contrast to the dancers in *Dance Innovations* (Coupland, 2013), Kathy had now rediscovered her dance ability instead of a loss of her dance skills over time. When we discussed the ideal dancer's body shape in the beginning of our memory-work discussions, we did not challenge its social construction. Now, from a more clearly defined feminist perspective, we regarded it as a socially created pressure rather than a taken-for-granted necessity. Mary Ann reiterated that women are, indeed, “obsessed about how they look,” but now considered that society “programs” us to think a certain appearance determines their self-worth. She found it “really detrimental to women.” Pirkko, Tamara, and Wendy reaffirmed that the ideal, feminine dancing body “reiterates it over and over again” (Tamara) throughout dance training and a dance career due to being “so pervasive in society” (Wendy) that it is seldom questioned. While the previous research indicates mature dancers being very aware of the “perfect” feminine dancing body and the pressures to conform to it, they did not indicate an open feminist awareness or resistance to its socially construction (e.g., Schwaiger, 2012; Coupland, 2013; Southcott and Joseph, 2020).

These feelings of freedom in movement were not necessarily readily available. Jodie remembered getting “panicky” while warming up before the performance. Wendy vividly described “walking through molasses, too slow, then too quick” before reaching quiet in her mind during the performance. Contrary to the cultural image of “empirical youthfulness” (Martin, 2017), these descriptions portray a deep trust on one's own mature body: “trust your body, you know the movement, I do,” Jodie emphasized. To enjoy movement, the dancers, nevertheless, needed ownership. As Tamara summarized: “As the dancer, I had permission to drive my own vehicle and for a ‘mature' body, this was so good and so necessary!” We viewed ourselves as highly capable performers, not aging dancers, an identity assigned to us by cultural expectations of youthfulness in dance as a performing art. This transformation was possible, we concluded, by the mutual support and understanding of each other as women. Our experiences during the actual performance further highlighted the shared appreciation of dancing with mature women who did not succumb to “normative decline and progressive disengagement from public display” (Schwaiger, 2012, p. 117).

### Once a Dancer Always a Dancer

This last theme presents our experiences of the final performance. It provided a culmination of our subversive transformation from self-doubting our abilities to confident performers drawing from the support of other skilled and confident mature dancers. While we had substantial experience, many of us had had a long performance break. For some, like Pirkko, entering the stage again was a source of significant anxiety as she was afraid of forgetting the movement sequences, a “cognitive” challenge shared by many mature dancers (e.g., Ali-Haapala et al., 2020; Southcott and Joseph, 2020). Jodie, however, sensed the tangible excitement of performing again:

I smile and open the theater door, “here we go.” I jump up on the stage and get ready to warm up the entire cast. It's exhilarating to sense the excitement, the joy and the buzz throughout the bodies in the space, that stage space.

Others felt “at home again.” For Kathy, it was “like riding a bike:” the habits of warming up, breathing, feeling ready to perform were readily recalled. Mary Ann affirmed: “Being on stage felt like yesterday, not 30 years ago…all came back instinctually. Feeling the black floor, enjoying the darkness, watching others in the previous dance zoom on and off with entrances and exits.” Her initial feelings of insufficient performance ability now changed as she considered that “once a dancer always a dancer. I like that.” Following Mary Ann, we did not have to resort to “performing our age” in the fear of making “spectacles of ourselves” (Schwaiger, 2012, p. 76) as substandard dancers or as less than we were previously—we were back, we believed, as legitimate dance artists.

Some aspects of the performance event had changed, however. These alternations further reflected our positive transformation and acknowledgment of maturity. One example was the preparation on the performance day. The external makeup was not as important as being prepared physically onstage (Kathy). While we observed the younger dancers glued to the mirrors in the dressing rooms, our preparation, instead, entailed our own individual rituals performed in isolation or with the group of others outside of the dressing rooms. For example, Mary Ann “gathered the 6 of us to…a tight circle, linking fingers, exchanging energies” close to our curtain call. These rituals gave us “security” (Wendy) as well as a chance to switch to the dancer/performer role among the multiple roles in our everyday lives. As Jodie explained: “I am an instructor, I am student, I am a mom, as well as a friend, but this particular day, I needed to concentrate on my role as a dancer.” At this point, Jodie no longer prioritized her family or her other roles but felt free to dedicate the day for dancing.

Tamara and Mary Ann now redefined the meaning of performance that previously seemed like “the whole purpose of rehearsing” (Tamara). In so doing, they began to challenge how dance as a performing art is constructed. Although Tamara still acknowledged the performance as “the culmination of the varied, long rehearsal experience…the icing on the cake,” she continued

it is more than the performing of the choreography…This performance was the culmination of the process of understanding, deciphering, struggling, contemplating and maybe even arguing with yourself or differing with others about how you should dance something just a certain way; struggling to find your peak ability to perform a sequence, or finding your timing in sync with another.

Mary Ann emphasized that instead of solely preparing “for such a fleeting finale,” our dancing together was “more about the process than the product and I can honestly say that each rehearsal was as fulfilling as the two performances—just in a different way.” She now explained that we danced “not so much for everyone else, but for ourselves.” In this sense, our examination of aging now resulted in further critical reflections of dance culture in general.

#### They Had My Back…and I Had Theirs

This final sub-theme reinforced that performing with other experienced dancers provided additional confidence and support with “a shared and unspoken understanding of each other as performers, dancers and women” (Mary Ann). Pirkko reflected: “They are all there, the dancers who will support me through this. They can be trusted, they will enjoy dancing.” Wendy now had let her previous worries go, because she “felt more confident” than in her previous performances: “I was amongst five other super dancers…A united group. What a difference that makes.” This trust and unity were, nevertheless, achieved to committed work to rehearse the piece. Jodie wrote: “All the blood, sweat, tears, scheduling and challenges are gone… We were committed, we worked hard, we trusted and respected our other fellow dancers and we battled through schedules, all for the ‘performance day.”'

Confident in our performance expression gained through the committed rehearsal process, we were now able to subvert even the social pressures of having to possess an ideal, feminine dancing body. Kathy, who was initially worrying about weight gain, now noticed how she was able to spend less time worrying about how she looked:

In the performance I was a little over weight, but I knew I had the physical ability, so I pushed through those little voices, and my insecurities, and…try not suffer with my self-worth, and say I am going to do it anyway…it didn't stop me.

Kathy now prioritized her technical ability as a mature dancer unlike the dancers in Schwaiger's (2012) study how tended to emphasize emotional expression instead of technicality they believed their bodies no longer sustained. The focus on physical ability, however, enabled Kathy to unmask the normalization of the ideal dancing body. Alongside Kathy, the other co-researchers also gained a different “purpose and identification as dancers” (Mary Ann) from our previous identities as young dancers. “We shared a powerful experience of the dance,” Mary Ann summarized, “and common identity as dancers regardless of age, ability or performance history. This was a wonderful feeling.”

Several of us remembered the “roaring applause” at the end of our performance. Kathy recalled: “There is a huge applause!! We did it and it was magical! We bow and run off with smiles on our faces! Good job ladies!” Instead of “sadly aging dancers” who were “unwatchable” (Coupland, 2013), we interpreted the audience reaction as praise for our impeccable performance expression. We left the rehearsal and performance of Initiation feeling “the buzz of dancing, the spirit of community, the positive vibe…from the performative bodies moving in artful ways” (Pirkko). While performing again solicited some anxiety, we emphasized an “unruffled”—excitement with an inner calm (Wendy)—meditative concentration on movement in a very supportive and safe group. “What a wonderful feeling” (Wendy) it was being confident in our abilities and artistic presentation. Challenging the invisibility of older women (dancers) was, as Mary Ann concluded, “magical.”

## Conclusion

Our memory-work analysis revealed that mature women dancers can subvert the idealization of youthfulness in dance. In this aspect, it aligned with findings by the previous scholars who have argued that contemporary women dancers, in particular, can resist the social construction of the aging body as frail, weak, and unsuitable for public performance (e.g., Schwaiger, 2005, 2012; Coupland, 2013; Brandstetter and Nakajima, 2017; Martin, 2017; Southcott and Joseph, 2020). The three main themes (“It Will Only Get Worse;” “It Can Be Magic;” and “Once a Dancer Always a Dancer”) illustrated our path to increased awareness of how ageism shaped our identities as dancing women as well as contemporary dance more broadly. The first theme (“It Will Only Get Worse”) reflected our initial acceptance that “empirical youthfulness” is necessarily expected when performing dance on public stage. We were afraid and fearful of not fulfilling such expectations having untrained, injured, or aging bodies and long absence from performing. These feelings stemmed from the current cultural and social context of aging and as such, tended to reproduce established ageist ideas of the feminine dancing body. However, the following two themes (“It Can Be Magic,” “Once a Dancer Always a Dancer”) demonstrated our growing subversion of the social construction of aging as we began to problematize youthfulness as a prerequisite for continued performance presence. We now questioned the need to conform with a certain body ideal and challenged the notion of having to be less physical as mature dancers. We enjoyed moving with a group of committed, hardworking dancers with similar age and ability. We found our dancing selves again in this cultural context without having to “mask aging” (Schwaiger, 2012, p. 76) by resorting age-appropriate movement vocabulary suitable for “old dancers” (Schwaiger, 2005, p. 112). Although we modified our choreography to our needs, we did not explicitly pare down the movement or our physical presence in favor of, for example, theatricality or emotional expression as reported by previous research (e.g., Schwaiger, 2005, 2012; Southcott and Joseph, 2020).

Through the memory-work, we reconstructed our identities as an experienced, committed group of dancers who brought respect, trust, hard work, commitment, confidence, and enjoyment to our performance. During this work, we increasingly connected our personal identity construction to social and cultural issues regarding aging and dance. When mature women dancers perform, we concluded, they openly challenge such a stereotypical image of the dancers. As we were able to verbalize these ideas through a memory-work research project, we also developed a conscious critical, feminist consciousness. While other researchers also interpreted mature dancers “challenging” the ageist evaluation of “older bodies” (Coupland, 2013) or “acquiring agency” (Southcott and Joseph, 2020, p. 604) by choosing to continue dancing, this “resistance” to aging was not necessarily openly expressed by the dancers themselves. Therefore, as memory-work co-researchers we were in a unique place to articulate the personal experiences and consciously connect them to the ideological construct of ageism. This presented the political value (Jansson et al., 2008) of our research: It documented the problematic process of ageism in dance and initiated social change by tapping into lived danced practices and our everyday experiences as mature women.

In this work, we hoped to raise critical age consciousness, albeit through micro action in a dance performance, by subverting stereotypical representations of aging, feminine bodies also in society at large (Martin, 2017). It expanded the previous dance studies literature on aging and professional dance by employing a feminist memory-work methodology that reduces the researcher-participant hierarchy by placing all the participants as co-researchers. The findings demonstrated that the process of choreographing and performance of contemporary dance emerged as a potential subversion of ageism. As such this work, raises critical age consciousness and we now feel empowered to continue critically contesting the cultural images of the dancing body with further choreographic works. Our research and dance practice are now designed to further highlight dance as a site for the subversion of the cultural norms of youthfulness. We conclude with Kathy's words: “We are doing a good job breaking all of those barriers and stereotypes and…keep doing what we are doing!”

## Data Availability Statement

The original contributions presented in the study are included in the article/supplementary materials, further inquiries can be directed to the corresponding author/s.

## Ethics Statement

The studies involving human participants were reviewed and approved by University of Alberta, Edmonton, Canada. The patients/participants provided their written informed consent to participate in this study.

## Author Contributions

PM analyzed the data and wrote the article. KM analyzed the data. TB, WG, MR, and JV were co-authors in the memory-work research. All authors contributed to the article and approved the submitted version.

## Funding

This work was supported by Social Sciences and Humanities Research Council, Canada Connection Grant (#611-2019-0314).

## Conflict of Interest

The authors declare that the research was conducted in the absence of any commercial or financial relationships that could be construed as a potential conflict of interest.

## Publisher's Note

All claims expressed in this article are solely those of the authors and do not necessarily represent those of their affiliated organizations, or those of the publisher, the editors and the reviewers. Any product that may be evaluated in this article, or claim that may be made by its manufacturer, is not guaranteed or endorsed by the publisher.
